# Advancing skin model development: A focus on a self-assembled, induced pluripotent stem cell-derived, xeno-free approach

**DOI:** 10.1177/20417314241291848

**Published:** 2024-11-05

**Authors:** Marla Dubau, Tarada Tripetchr, Lava Mahmoud, Vivian Kral, Burkhard Kleuser

**Affiliations:** Department of Pharmacology and Toxicology, Institute for Pharmacy, Freie Universität Berlin, Berlin, Germany

**Keywords:** Hair-follicle derived cells, reprogramming, induced pluripotent stem cells, keratinocytes, fibroblasts, self-assembled skin model, xeno-free approach

## Abstract

The demand for skin models as alternatives to animal testing has grown due to ethical concerns and the need for accurate substance evaluation. These alternatives, known as New Approach Methodologies (NAMs), are increasingly used for regulatory decisions. Current skin models from primary human cells often rely on bovine collagen, raising ethical issues. This study explores self-assembled skin models (SASM) as a new method, utilizing hair follicle-derived keratinocytes reprogrammed into induced pluripotent stem cells (iPSC) and differentiated into fibroblasts and keratinocytes. The model relies on the ability of fibroblasts to secrete collagen to produce a xeno-free dermal layer and on the differentiation of keratinocytes to create a functional epidermal layer. These layers exhibited confirmed metabolic activity and the capability to withstand test substances. The successful development of SASM underscores the significance of accurate alternatives in dermatological research, providing an ethical and reliable option for substance evaluation and regulatory testing.

## Introduction

The need to develop alternatives to animal testing in skin research is driven by both ethical considerations and the commitment of the scientific community to advance humane and reliable research methods. Recognition of the limitations and ethical concerns associated with traditional animal experimentation has led to the 3Rs principles of replacement, reduction and refinement of animal experiments, a legal requirement in the European Union. This effort has been supported by the US Food and Drug Administration (FDA). These newly developed alternative methods are referred to as New Approach Methods (NAMs). NAMs involve any technology, methodology, approach, or combination thereof that can provide data for chemical hazard and risk assessment without the use of animals. This includes *in silico, in chemico, in vitro*, and *ex vivo* approaches.^1–3^ Current skin models primarily use animal-derived components, such as bovine collagen or similar scaffolds, to embed fibroblasts, and construct an artificial dermal layer. However, the use of these components raises ethical concerns and introduces potential risks associated with both interspecies and batch-to-batch variability.^4^

Fibroblasts play a crucial role in synthesizing the extracellular matrix (ECM) to form connective tissue. This involves depositing various components, including fiber- and sheet-forming collagens, proteoglycans, elastin, fibronectin, microfibrillar proteins, and laminins, which collectively form the ECM.^5,6^ It is worth noting that fibroblasts from different sources have varying extracellular matrix profiles. Fibroblasts can be activated by various growth factors and cytokines. One of the most important factors is TGF-ß.^7,8^

Traditional reconstructed human skin models use fibroblasts and keratinocytes, either from primary cells or established cell lines, to mimic the dermal and epidermal layers, respectively, with the aim of replicating the structural and functional characteristics of physiological skin.^9,10^

Both cell lines and primary cells have been crucial in developing reconstructed human skin models, providing valuable insights into skin biology. However, they lack the genetic and phenotypic diversity found in natural human populations.^11^ Induced pluripotent stem cell (iPSC)-derived cells can provide a more versatile and physiologically relevant model.^12^ This model can be tailored to reflect a wider range of genetic backgrounds and disease states, enhancing the predictive accuracy of skin models for research and therapeutic purposes.

In this study, xeno-free, self-assembled full-thickness skin equivalents that feature both dermal and epidermal layers have been developed. This facilitates intercellular communication between iPSC-derived keratinocytes and fibroblasts. Furthermore, the role of *L*-ascorbic acid in fibroblast differentiation was explored and the collagen production under varying conditions was analyzed. The study suggests that both iPSC-derived fibroblasts and normal human dermal fibroblasts (NHDF) are suitable for constructing these models. However, iPSC-FB offer advantages in terms of availability, genetic diversity, and phenotypic consistency compared to NHDF.

Moreover, the SASM exhibited strong metabolic activity and responded to 2,4 dinitrochlorobenzene (DNCB), a potent skin sensitizer through the secretion of inflammatory cytokines such as IL-8 and IL-1β. These findings highlight the potential of SASM as a possible model for studying skin responses to various stimuli, without the ethical and biological complications associated with using animal-derived materials.

## Materials and methods

### Isolation and cultivation of hair-follicle-derived keratinocytes and fibroblasts

For the isolation of HFDK and HFDF, the method of Loewa et al.^13^ was used as guidance. About 30–40 hair follicles were plucked from male and female donors aged between 25 and 35 years (ethical approval EA2/006/15) and carefully placed onto an insert membrane of a transwell plate coated with a poly-D-Lysine hydrobromide solution (Sigma-Aldrich, Munich, Germany). About 16 h prior to the experiment, postmitotic 3T3-J2 fibroblasts were seeded as feeder cells onto the basal side of the insert. Up until reaching 70% of confluency, the hair follicles were cultured in outer root sheath (ORS) medium (ORM+), containing 64,5% Dulbecco’s Modified Eagle Medium (DMEM) and 21,5% Ham’s F12 Nut mix with GlutaMax™ (both from Gibco, Waltham, MA, USA), 10% fetal bovine serum (FBS), 100 U/mL penicillin/streptomycin, 0.4 µg/mL hydrocortisone, 0.1 nM cholera toxin, 10 ng/mL epidermal growth factor, 0.18 mM adenine, 4 mM *L*-glutamine, 2 nM liothyronine (all from Sigma-Aldrich, Munich, Germany), and 5 µg/mL insulin (Roche, Basel, Switzerland). After approximately 3 weeks, the outgrown cells from the ORS were harvested by trypsinization and used for further cultivation. Therefore, a detachment time-selection strategy was employed, a 5-min treatment with trypsin-EDTA (Sigma-Aldrich, Munich, Germany) to obtain HFDF and 10-min treatment with trypsin-EDTA to obtain HFDK. HFDF were further cultivated in fibroblast growth medium (FGM) containing DMEM (Sigma-Aldrich, Munich, Germany) supplemented with 10% FBS, 1% *L*-glutamine, and 1% penicillin/streptomycin for up to six passages. HFDK were cultured on PureCol EZ gel (Merck, Darmstadt, Germany) coated plates in keratinocytes growth medium 2 (KGM2; PromoCell, Heidelberg, Germany) for up to six passages.

### Reprogramming of HFDK into iPSC

HFDK were cultured in KGM-2. Two days prior to the transduction, 1.5 × 10^5^ HFDK (passage 4) were seeded into each well of a six-well plate. The reprogramming of HFDK was done using the CytoTune™-iPS 2.0 sendai reprogramming kit in accordance to the manufacturer’s protocol (ThermoFisher Scientific, Waltham, USA). The Sendai virus vectors contain transgenes designed to induce the expression of hOct3/4, hSox2, hKlf4, and hc-Myc. After the transduction, these four viral vectors will initiate reprogramming through overexpressing these four genes in the cells. On day 0, the CytoTune 2.0 Sendai tubes were removed from −80°C storage, the KGM-2 was aspirated from the wells containing HFDK and each well was washed twice with 2 mL PBS. The reprogramming virus mixture was prepared according to the manufacturer’s instructions. Briefly, 100 µL KOS, 100 µL hc-Myc, 66 µL hKLF4, and 20 µL Y27632 were resuspended in 40 mL of KGM-2 and 1 mL was added to each well within 5 min. For reproducibility, the applied multiplicity of infection (MOI) was 3.6:3.6:2 for hKlf4-hOct3/4-hSox2: hc-Myc: hKlf4, respectively. The cells were incubated overnight at 37°C and 5% CO_2_. Subsequently, 24 h after transduction, the wells were washed once with 2 mL PBS and cultured in fresh KGM-2. Cells were cultured in KGM-2 for seven more days, with a medium change every other day. On day 7, the cells were passaged onto vitronectin (ThermoFisher Scientific, Waltham, USA)-coated plates with a final coating concentration of 0.5 µg/cm^2^. From day 8 to 28 the cells were cultured in CTS™ Essential 8™ Medium (ThermoFisher Scientific, Karlsruhe, Germany) supplemented with 10 µM StemMACS™ Y27632 (Miltenyi Biotech, Bergisch Gladbach, Germany). Fully differentiated colonies, exhibiting the typical iPSC-colony morphology with smooth edges of the colonies, as well as a high nucleus-cytoplasm ratio, were manually picked to ensure the selection of fully reprogrammed cells only and transferred onto vitronectin-coated 6-well-plates from day 29 on.

### Culturing NHEK and NHDF

NHEK and NHDF were obtained from the juvenile foreskin of male donors <10 years of age according to protocol from another study.^14^ NHEK were cultured in KGM-2 on collagen-coated plates; PureCol EZ Gel at a concentration of 5 mg/mL was used for the coating process and NHEF were cultured in FGM. NHEK and NHDF were used up to six passages. These cells were used as a positive control for their iPSC-generated counterparts.

### Differentiation of iPSC into fibroblasts

12-well plates were coated with 5 µL gelatin (Sigma-Aldrich, Munich, Germany) solution/cm^2^ and stored at 4°C. Embryoid bodies (EB) were generated out of iPSC-colonies in passage 12 using the hanging drop method with 4000 cells per 20 µL drop, CTS™ Essential 8™ Medium with 4 mg/mL polyvinyl alcohol (Sigma-Aldrich, Munich, Germany) was selected. After 48 h, the EB were collected and transferred onto ultra-low-attachment 6-well plates (Corning Inc., Corning, NY, USA) (10 EB per 1 well) and cultured for another 48 h. Afterwards, the EB were transferred onto gelatine-coated plates (Sigma-Aldrich, Munich, Germany; day 0) and cultured in fibroblast differentiation mix 1 (FDM1) for 96 h with a medium change every 24 h. FDM1 consisted of DMEM high glucose supplemented with 0.3 mM *L*-ascorbic acid (CAS: 50-81-7, purity >98%, Sigma-Aldrich, Munich, Germany), 5 µg/mL insulin, 0.18 mM adenine, 10 ng/mL epidermal growth Factor (EGF), and 10% FBS. On day 4–6, the FDM1 was additionally supplemented with 0.5 nM bone morphogenetic protein 4 (BMP4; Miltenyi Biotech, Bergisch Gladbach, Germany). On day 7, the medium was changed to fibroblast differentiation mix 2 (FDM2), a 1:1 mixture of DMEM high glucose and F12, supplemented with 0.3 mM *L*-ascorbic acid, 10% FBS and 1% nonessential amino acids (ThermoFisher Scientific, Waltham, USA). The cells were cultured in this medium for 7 days and started to express a fibroblast-typical spindle shape. On day 14, the cells were trypsinized with TrypLE™ Select for 5 min (Thermo Fisher Scientific, Karlsruhe, Germany) and seeded onto non-coated T75 cm^2^ flasks and cultured in FDM1 for 7 days. Afterwards, the iPSC-FB were passaged onto collagen-coated plates and either cultured in FGM, or in a serum-free alternative, Cnt-Prime Fibroblast ECM Medium (CELLnTEC, Bern, Switzerland). To evaluate the necessity of *L*-ascorbic acid during the differentiation process, a differentiation under identical conditions of iPSC into iPSC-FB with the only difference being the complete absence of *L*-ascorbic acid throughout the entire process was conducted.

### Differentiation of iPSC into keratinocytes

For the differentiation of iPSC into keratinocytes, the protocol from other studies were adapted.^14,15^ iPSC were cultured on vitronectin-coated 6-well-plates until they reach a confluency of 70%. EB were generated out of iPSC in passage 14 as described previously. The EB were transferred onto previously coated geltrex-collagen plates. For the coating of one 6 cm petri dish, a mixture of 5 mL 1:1 DMEM/F12 mix with 50 µL growth factor reduced geltrex (ThermoFisher Scientific, Waltham, USA) and 50 µL PureCol EZ Gel was added into the dish and incubated at 37°C at 5% CO_2_ for 1 h. On day 0 of the differentiation, 15 EB were transferred onto one 6 cm dish and cultured in 4 mL KGM-2 supplemented with 1 µM final working concentration of retinoic acid (Sigma-Aldrich, Munich, Germany), 25 µg/mL BMP4 and 2 mM *L*-glutamine and incubated for 48 h. On day 2, the medium was aspirated from the dishes, and 4 mL of fresh medium was added. After another round of 48 h incubation, the medium was changed to KGM-2 with 2 mM *L*-glutamine for the next 10 days, with a medium change every other day. On day 14, the plates were washed with 3 mL PBS and passaged onto collagen-coated plates in 4 mL CnT Prime Epithelial Proliferation Medium (CELLnTEC, Bern, Switzerland). Most cells start to express an epithelial phenotype at this point. After 7 days on the collagen plates, the rapid attachment method was performed to increase the purity of the differentiated cells. Therefore, 10 cm petri-dishes were coated with a mixture of PureCol EZ Gel (3 µg/mL) and collagen IV (7 µg/mL; Merck, Darmstadt, Germany). The cells were were trypsinized with TrypLE™ Select for 8 min (Thermo Fisher Scientific, Karlsruhe, Germany) and transferred onto the collagen I (5 mg/mL)-collagen IV (0.3 mg/mL)-coated plates and incubated for 30 min at room temperature. The fully differentiated cells were more likely to adhere to the collagen I-collagen IV-coated plate during this process. After 30 min, the medium was aspirated, the cells were trypsinized and resuspended in fresh Cnt Prime Epithelial Proliferation medium and added on freshly coated collagen I plates.

### RNA extraction, cDNA synthesis, and qPCR

Total RNA was extracted using RNeasy^®^ Kit (Qiagen, Hilden, Germany) following the manufacturer’s instructions, and RNA purity was assessed using a NanoDrop spectrophotometer (NanoPhotometer^®^ N60 UVVis spectrophotometer (Implen, München, Germany)). cDNA synthesis was performed according to the manufacturer’s instruction with 40 µg of RNA using first strand cDNA synthesis Kit (ThermoFisher Scientific, Karlsruhe, Germany) qPCR was conducted using Human Pluripotent Stem Cell Trilineage Differentiation qPCR array (Stemcell Technologies, Lyon, France) on a LightCycler^®^480 II (Roche, Mannheim, Germany) using qPCR Master Mix (Stemcell Technologies, Lyon, France), with each reaction containing 5 µL of the qPCR Master Mix, 1 µL cDNA, and nuclease-free water in a 10 µL total volume. The cycling conditions were 95°C for 20 s, followed by 45 cycles of 95°C for 1 s, and 60°C for 20 s. The instruction from the manufacturer of the qPCR array was strictly followed. Relative gene expression of iPSC to HFDK was calculated using the 2^−ΔΔCt^ method with β-actin as an internal control. All reactions were performed in triplicate and melt curve analysis confirmed product specificity.

### Senescence detection in iPSC

To detect cellular senescence in iPSC, the cells were prepared following a standard protocol optimized for flow cytometry analysis (CellEvent™ Senescence Green Flow Cytometry Assay Kit, ThermoFisher Scientific, Karlsruhe, Germany). A negative control was prepared by incubating the iPSC in CellEvent™ Senescence buffer in the absence of a senescence-inducing agent. iPSC from passage 6 to 23 were harvested and resuspended at a concentration of 0.5 × 10⁶ cells per mL in culture media. After harvesting, cells were centrifuged, and the pellets were resuspended in 400 µL of fixation solution (2% PFA) and incubated on ice for 15 min, in the dark. Subsequently, cells were centrifuged and washed with 400 µL of 1% BSA in PBS to remove the fixation solution. The cell pellets were then resuspended in 100 µL of the senescence detection working solution, with negative control cells resuspended in 100 µL of CellEvent™ Senescence buffer and incubated for 1 h at 37°C without CO₂, protected from light. Following incubation, the cells were centrifuged, washed with 600 µL of 1% BSA in PBS, and resuspended in 300 µL of 1% BSA in PBS and analyzed by flow cytometry.

### Protein extraction and western blot

iPSC (passage 15) and iPSC-FB (passage 6) cells were harvested, and cell pellets were resuspended in 125 µL RIPA buffer supplemented with PBS, protease inhibitors (cOmplete™, Roche, Basel, Switzerland), and phosphatase inhibitors (PhosSTOP™, Roche, Basel, Switzerland). The suspension was vortexed briefly and lysed using an ice-cooled ultrasonic bath for 10 min. The lysate was centrifuged at 12000 × *g* for 30 min at 4°C, and the supernatant containing solubilized proteins was collected. Protein samples (20 µg) were mixed with 6× Laemmli buffer, denatured at 95°C for 5 min, and separated by 10% SDS-PAGE (Bio-Rad, Munich, Germany) at 200 V for 1 h 15 min. Proteins were transferred onto nitrocellulose membranes, blocked with 5% BSA for 1 h, and incubated overnight at 4°C with primary antibodies: Nanog (1:1000), Lin28a (1:1000), and TRA-1-81 (1:1000). All the markers were from Biologend, San Diego, USA. Blots were washed and incubated with HRP-conjugated secondary anti-mouse (Cell Signaling, Frankfurt/Main, Germany) antibody for 1 h. Detection was performed using ECL substrate (Bio-Rad, Munich, Germany), and bands were visualized with the ChemiDoc MP Imaging System.

### Characterization of iPSC, iPSC-FB, iPSC-KC, HFDK, HFDF, NHEK, and NHDF by immunofluorescence staining

2 × 10^4^ cells were seeded into a one chamber slide (Sarstedt, Nümbrecht, Germany). iPSC were cultured in CTS™ Essential 8™ Medium on vitronectin-coated chamber slides for 48 h. iPSC-KC, HFDK and NHEK were cultured in 3 mL Cnt Prime Epithelial Proliferation medium for 24 h, iPSC-FB, HFDF, and NHDF were cultured in FGM and another set of the three types of fibroblasts was cultured in Cnt Prime ECM Fibroblast medium for 24 h. Subsequently, the medium was aspirated, and the chamber slides were washed twice with 2 mL PBS before permeabilizing the cells with 0.5% (V/V) Triton X-100 (Sigma-Aldrich, Munich, Germany) in PBS for 15 min. After a washing-step with PBS/BSA (Sigma-Aldrich, Munich, Germany)/Tween20 (Carl Roth, Karlsruhe, Germany), the cells were blocked with goat serum (1:20; ThermoFisher Scientific, Waltham, USA) for 30 min at room temperature before adding the primary antibody to the cells. iPSC were stained with anti-Ms X SSEA-4, Rb X Nano, human (both from Merck, Darmstadt, Germany), anti-TRA-1-60 Monoclonal Antibody (ThermoFisher Scientific, Karlsruhe, Germany), Sox2 Antibody, anti-human/mouse, REAfinity™ or Oct3/4 Isoform A Antibody, anti-human/mouse, APC, REAfinity™ (both from Miltenyi Biotec GmbH, Bergisch Gladbach, Germany). The fibroblasts, previously cultured in FGM or Cnt Prime ECM Fibroblast medium respectively, were incubated with either anti-vimentin-FITC, anti-collagen I-PE, or anti-collagen IV-PE (all from Miltenyi Biotec GmbH, Bergisch Gladbach, Germany) overnight. The keratinocytes were incubated with anti-cytokeratin 10-PE or anti-cytokeratin 14-FITC (both from Miltenyi Biotec, Bergisch Gladbach, Germany) or anti-cytokeratin 1 (Abcam, Cambridge, UK) overnight. All dilutions were done according to the instructions of the manufacturer. For the cells stained with SSEA4 and Tra-1-60, a secondary mouse anti-goat antibody Alexa Fluor 488 (Abcam, Cambridge, United Kingdom) at a dilution of 1:400 and for the cells stained with Nano-G and cytokeratin 1, a secondary goat anti-rabbit antibody Alexa Fluor 555 (ThermoFisher Scientific, Karlsruhe, Germany) at a dilution of 1:500 was added the next day for 1 h. Afterwards, one drop of ProLong™ Gold antifade mountant with 4′,6-diamidino-2-phenylindole (DAPI, ThermoFisher Scientific, Karlsruhe, Germany) was added onto the cover slides before adding a glass cover slide. The cells were analyzed with a KZ-8001 Fluorescence microscope (Keyence, Neu-Isenburg, Germany).

### Corrected total cell fluorescence of fibroblasts and keratinocytes from the three different cell sources

To determine the level of cellular fluorescence of fibroblasts and keratinocytes from fluorescence microscopy images, corrected total cell fluorescence (CTCF) measurement was performed. Fluorescent images of NHDF, HFDF, iPSC-FB, NHEK, HFDK, and iPSC-KC were acquired using the Keyence KZ-8001 fluorescence microscope, at a magnification of 40×, an identical exposure time for all the fibroblasts from different sources and all the keratinocytes from different sources. Raw fluorescence images were processed using the ImageJ software. Regions of interest (ROIs) were defined around individual cells using manual selection. For each cell type, 20 cells were selected per repetition as ROIs. Background fluorescence was determined from non-cellular regions and subtracted from the fluorescence intensity within the ROIs to account for background noise. The CTCF was calculated using the following formula:

$$CTCF\;=\;Integrated\;Density-\left(\begin{array}{l} Area\;of\;Selected\;Cell\;\\ \times \;Mean\;Fluorescence\;of\;\\ Background\;readings \end{array}\right)$$

CTCF values for 20 cells were determined, and statistical analyses were performed using the ordinary One-way ANOVA test in GraphPad Prism.

### Determination of collagen levels within the different fibroblasts

In order to investigate the differences of collagen secretion from fibroblasts of different sources, a soluble collagen quantification assay kit (Sigma-Aldrich, Munich, Germany) was used. The kit can be used to quantify soluble collagen types I, II, III, IV, and V from adherent cells and secreted collagen from cell culture supernatant. About 1 × 10^6^ NHDF, HFDF, iPSC-FB, and iPSC-FB differentiated without *L*-ascorbic acid were seeded into T75 cm^2^ flasks. The cells were cultured in either FGM, FGM supplemented with 0.3 mM *L*-ascorbic acid or Cnt-Prime Fibroblast ECM Medium for 5 days. For the sample preparation as well as the assay procedure, the manufacturer’s instructions were followed. The fluorescence intensity was measured with the TECAN (infinite M200 pro, Tecan Group Ltd., Männedorf, Switzerland) at an excitation wavelength of 375 nm and an emission wavelength of 465 nm. To determine the amount of soluble collagen type I, the most prominent type of collagen within the human skin, an additional ELISA assay with the Human Collagen Type I ELISA Kit (Abcam, Cambridge, United Kingdom) was performed. The cell culture supernatant of NHDF, HFDF, and iPSC-FB cultured in Cnt-Prime Fibroblast ECM Medium for 5 days was collected. For the ELISA assay, the instruction of the manufacturer was followed.

### Generation of self-assembled skin model

To build the SASM, iPSC-FB were cultured in T75 cm^2^ flasks in Cnt-Prime Fibroblast ECM Medium. After trypsinization with TrypLE™ Select (Thermo Fisher Scientific, Karlsruhe, Germany) the cells were resuspended in Cnt-Prime Fibroblast ECM Medium with 5000 cells per 500 µL. About 500 µL of this cell suspension was added into each insert of a 12-well plate (Corning, CLS3460). About 1.5 mL medium was added into each well. The medium in the insert and in the well were changed every other day for 14 days. On day 14, iPSC-KC in passage 6 were added on top of the solidified extracellular matrix. About 0.9 × 10^6^ iPSC-KC were added into each insert. Therefore, iPSC-KC were trypsinized and resuspended CnT Prime Epithelial Proliferation Medium (0.9 × 10^6^ iPSC-KC per 500 µL). The medium in the wells was changed to CnT Prime Epithelial Proliferation Medium. After 24 h at 37°C and 5% CO_2_, an airlift was performed to allow the keratinocytes to be cultured in an air-liquid interface to allow differentiation of the keratinocytes. The medium in the wells was changed to Cnt-Prime Airlift (CELLnTEC, Bern, Switzerland) and the SASM were cultured in this medium for 10 days with a medium change every other day.

### Histology and immunofluorescence staining

Upon the completion of the cultivation, the membrane with the self-assembled skin-models was cut out of the insert and embedded in Frozen section compound (Leica Biosystem, Wetzlar, Germany) in a 24-well-kryotray before being snap frozen in liquid nitrogen and stored at −80°C. The frozen SASM were sectioned vertically with a Leica CM1510 Cryotome (Leica Biosystems, Wetzlar, Germany) at thickness of 7 µm and placed onto Poly-Prep microscope slides (Merck, Darmstadt, Germany). For both, H&E staining and immunofluorescence staining (IF-staining), the slides with the skin model sections were fixed in a first step by using 4% Roti-Histo-Fix (Carl Roth, Karlsruhe, Germany). The hematoxylin and eosin staining was performed according to standard protocols with all materials from Carl Roth, Karlsruhe, Germany. After fixation, the skin model-sections were permeabilized for 15 min with 0.5% (V/V) Triton-X in PBS for the IF-staining and blocked with 1:20 Goat serum in PBS for 30 min to reduce non-specific antibody binding. The sections were incubated overnight at 4°C in the dark with either anti-cytokeratin 10-Alexa Fluor ^®^ 647 (Abcam, Cambridge, UK), Anti-cytokeratin 14 REAfinity™ (Miltenyi Biotech, Bergisch Gladbach, Germany), Anti-Loricrin (Abcam, Cambridge, UK), anti-Collagen IV (Miltenyi Biotech, Bergisch Gladbach, Germany), anti-vimentin (Miltenyi Biotech, Bergisch Gladbach, Germany), anti-filaggrin, anti-claudin, or anti-Laminin (all three from Invitrogen, Carlsbad, USA). The next day, the slides were incubated with a secondary antibody, either Goat Anti-Rabbit Alexa Fluor 555 or Mouse Anti-Goat Alexa Fluor 488 for 1h at room temperature in the dark. After washing with PBS/BSA/Tween, a drop of ProLong™ Gold Antifade Mountant with DAPI was added. Pictures were taken with a KZ-8001 Fluorescence microscope.

### Flow cytometry analysis

iPSC, iPSC-FB, iPSC-KC, NHDF, and NHEK were harvested and divided into FACS tubes at 2 × 10^5^ cells per tube. After washing twice with Cells Staining Buffer (Biolegend, San Diego, USA) staining was performed. iPSC, iPSC-FB, and NHDF were stained with anti-CD10-APC (Biolegend), anti-CD44-eFluor^®^ 450 (Thermo Fisher Scientific, Karlsruhe, Germany), anti-CD90-PerCP (Biolegend, San Diego, USA), anti Tra-1-60-PE (Biolegend, San Diego, USA), and iPSC, iPSC-KC as well as NHEK were stained with anti-CD29-FITC, anti-CD49f-PE (both Miltenyi Biotech, Bergisch Gladbach, Germany), and Tra-1-60 and incubated with the respective markers for 30 min on ice, in the dark. All antibodies were diluted according to the manufacturer’s instructions. After this incubation, the excess marker was washed off and the marker expression was measured using CytoFLEX (Beckman Coulter, Krefeld, Germany). Unstained cells served as an unstained control and UltraComp eBeads™ Compensation Beads (Thermo Fisher Scientific, Karlsruhe, Germany) were used to achieve a compensation. The marker expression was analyzed using FlowJo version 10.8.01 (BD Bioscience, San Jose, USA).

### SASM treatment and cytokine secretion measurement

SASM were topically treated with 5 µM DNCB (CAS: 97-00-7, purity >99%, Sigma-Aldrich, Munich, Germany), or 50 ng/mL tumor necrosis factor-alpha (TNF-α) and 50 ng/mL IL-1β (both from Miltenyi Biotech, Bergisch Gladbach, Germany) as a positive control and incubated for 24 h in the cell incubator with 37°C and 5% CO_2_. An untreated control (UT) was included to establish baseline cytokine levels. Following treatment, IL-8 and IL-1β secretion were assessed using an ELISA kit (both from Biolegend, San Diego, USA) according to the manufacturer’s instructions.

### SASM viability assay

The viability of the SASM was determined using the 3-(4,5-dimethylthiazol-2-yl)-2,5-diphenyltetrazolium bromide (MTT) assay. In addition to the UT and DNCB-treated samples, a vehicle control (0.1% dimethyl sulfoxide (DMSO) in medium) and a positive control (100% DMSO; CAS: 67-68-5, purity >99,8%) were included. All chemicals were obtained from Sigma-Aldrich (Munich, Germany). A 5 mg/mL MTT stock solution was diluted 1:10 with Cnt-Prime Airlift medium. SASM inserts and wells were washed twice with PBS before adding 500 µL MTT solution to the inserts and 1000 µL to each well. The plates were incubated for 3 h at 37°C and 5% CO2. Afterwards, the MTT solution was aspirated, and 500 µL of 100% DMSO was added to the inserts and 1000 µL to the wells. The plates were shaken for 5 min at 600 rpm, and the absorbance was measured at 570 nm using a plate reader.

## Results

### HFDK can be fully reprogrammed into iPSC

**Figure 1.**
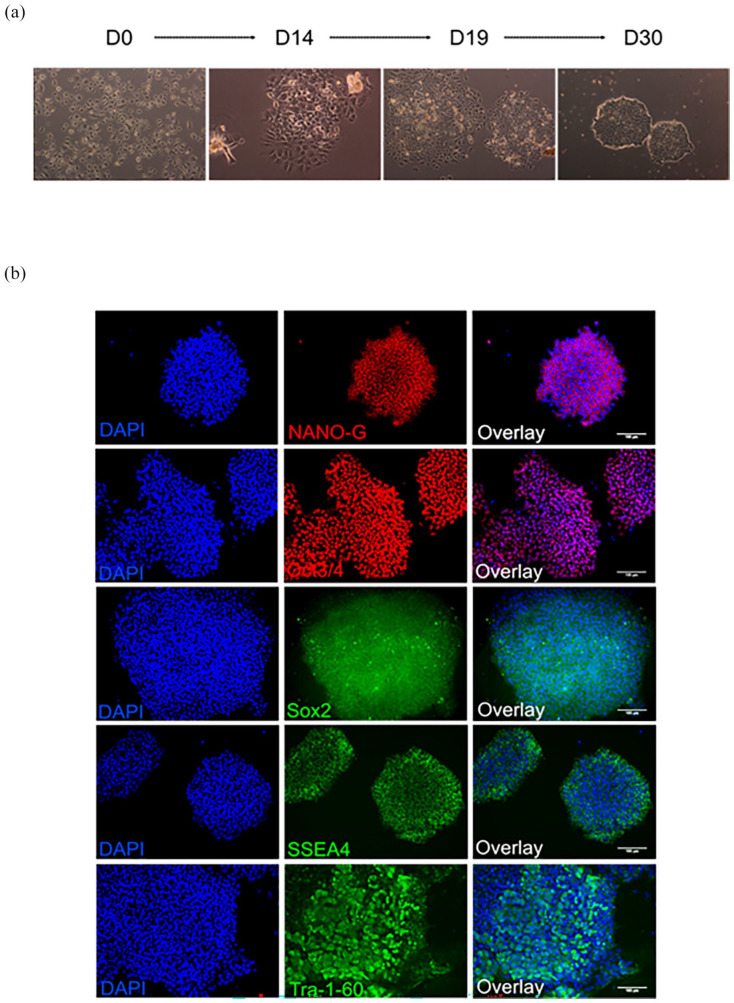
Representative microscopic images of hair follicle-derived keratinocytes (HFDK) reprogrammed into induced pluripotent stem cells (iPSC): (a) Microscopic images depict cell morphology changes at different stages (days 0, 14, 19, and 30). (b) microscopic images from day 30 immunofluorescence staining against stem cell markers (NANO-G, OCT3/4, Sox2, SSEA4, and Tra-1-60). DAPI served as nuclei counterstaining. Scale bar: 100µm (shown in overlay images). *N* = 5.

HFDK were successfully isolated and reprogrammed into iPSC utilizing Sendai-virus vectors for the delivery of Yamanaka factors (Oct3/4, Sox2, Klf4, and c-Myc). At day 0 pre-infection, HFDK retained their keratinocyte-like morphology, characterized by a pebble shape and a loose adherence pattern. By day 14 post-transfection, a significant morphological transformation was observed, with colonies developing a denser structure indicative of stem cell properties, including tightly packed cells with large nuclei that nearly filled the entire cell volume, yet the overall cell size remained akin to that of the original keratinocytes. By day 19, these colonies had expanded in size, with a noticeable reduction in cell size and increased colony compactness. At day 30 post-infection, the emergence of colonies resembling typical stem cell morphology comprising small, tightly clustered cells was evident (Figure 1(a)). Immunofluorescence staining further validated the stem cell identity of the colonies, demonstrating expression of pluripotency markers NANO-G, Oct3/4, Sox2, SSEA4, and Tra-1-60. This progression confirms the successful reprogramming of HFDK into iPSC and illustrates the dynamic morphological changes accompanying cellular reprogramming, culminating in the acquisition of stem cell characteristics.

### After reprogramming, iPSC express typical pluripotency markers

**Figure 2.**
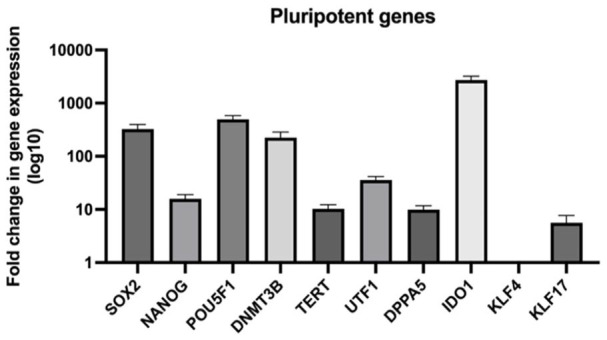
Quantitative PCR (qPCR) analysis of pluripotency gene expression in iPSC relative to HFDK. Relative fold change in gene expression of pluripotency markers in induced pluripotent stem cells (iPSC) compared to hair follicle-derived keratinocytes (HFDK) as the control. Gene expression levels were normalized to a housekeeping gene β-Actin, and the fold changes were calculated using the comparative 2-ΔΔCT method, with results displayed on a logarithmic scale (log10). The analyzed genes include SOX2, NANOG, POU5F1, DNMT3B, TERT, UTF1, DPPA5, IDO1, KLF4, and KLF17. Data represent the mean ± *SD, N* = 3.

After reprogramming HFDK into iPSC, we analyzed the resulting iPSC colonies for phenotypic characteristics by examining pluripotency and stem cell transcription markers. The expression levels of pluripotency markers, including SSEA-4, Tra-1-60, Oct 3/4, and Sox2, as well as the presence of intracellular NANO-G were assessed using immunofluorescence staining (Figure 1(b)). It is important to note that there was a significant increase in the expression of these markers. Additionally, we assessed the expression levels of keratinocyte markers CK10 and CK14 to confirm the successful reprogramming from the initial HFDK. No significant detection of keratinocyte markers was observed. Furthermore, the qPCR analysis comparing the fold change in pluripotency gene expression of iPSC to the HFDK was conducted. The result showed that iPSC have upregulated SOX2, NANOG, POU5F1, DNMT3B, TERT, UTF1, DPPA5, IDO1, and KLF17 after reprogramming indicating the pluripotency characteristics of the cells, confirming the success of the reprogramming of HFDK to iPSC (Figure 2).

### Senescence detection in iPSC across passages

**Figure 3.**
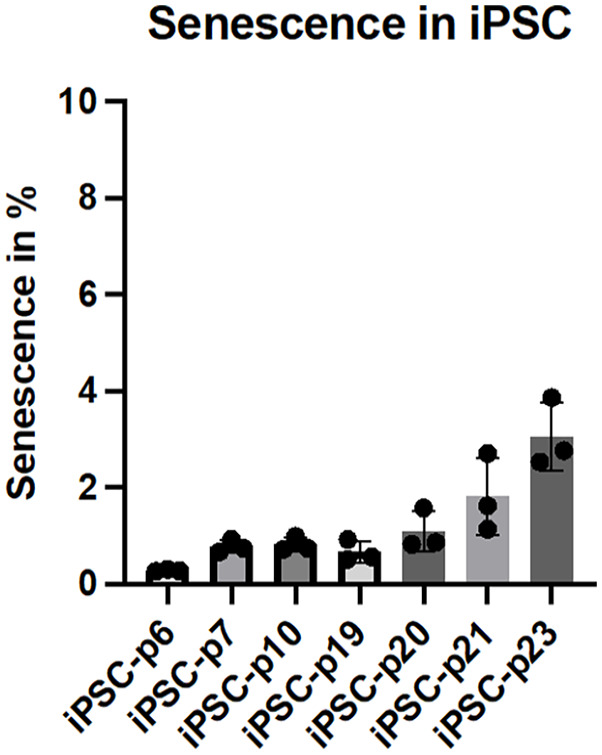
Senescence detection in iPSC across different passages. Induced pluripotent stem cells (iPSC) from passage 6 to 23 were analyzed for signs of senescence using a fluorescent substrate that is activated by senescence-associated β-galactosidase (SA-β-gal) activity, a key marker of cellular senescence. The percentage of senescent cells was quantified and is presented as mean ± *SD, N* = 3.

To assess the effect of increasing passages on senescence in iPSC, the senescence levels in iPSC were analyzed using the CellEvent™ Senescence assay followed by flow cytometry. The results show that despite the increase in passage number, the proportion of senescent iPSC remains consistently low. Even at higher passages (passage 23), the senescence levels in iPSC remained below 4%, with only minor fluctuations observed between earlier (passage 6), and later passages. This indicates that iPSC maintain their low levels of senescence across multiple passages, suggesting a robust ability to preserve their proliferative and non-senescent state over extended culture periods (Figure 3). These results demonstrate that iPSC do not undergo significant increases in senescence, even after prolonged expansion, further highlighting their stability and suitability for long-term culture.

### Characterization of iPSC derived fibroblasts in comparison to hair-follicle and human foreskin derived fibroblasts and influence of *L*-ascorbic acid on the differentiation of iPSC into fibroblasts

**Figure 4.**
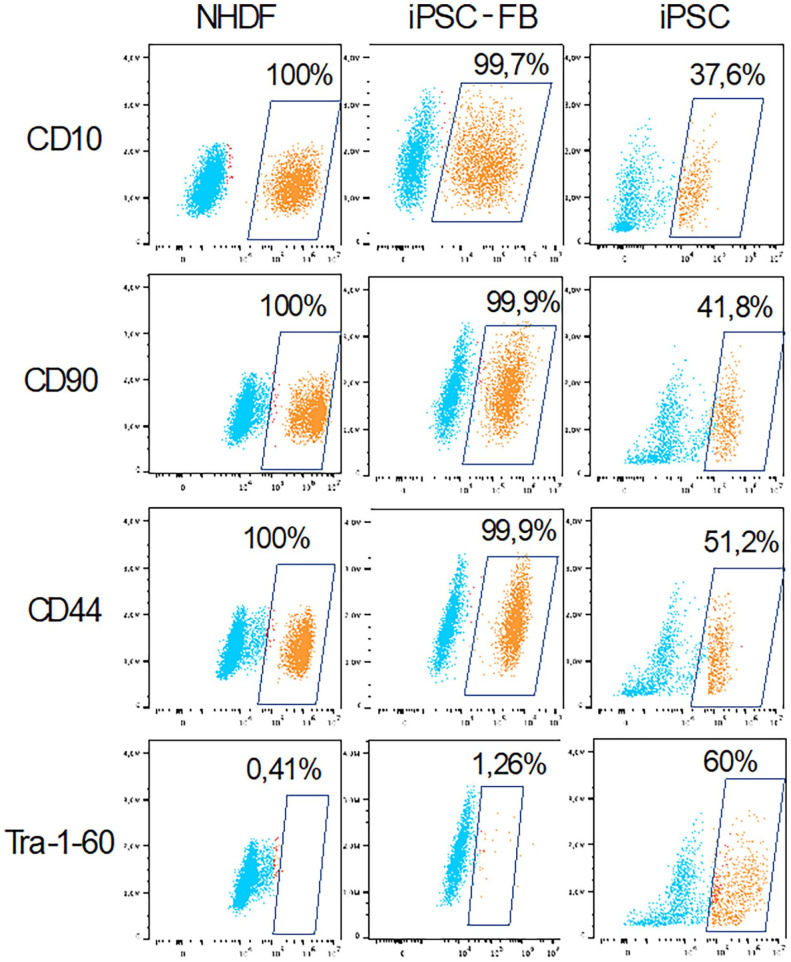
Representative dot plots illustrate the characteristics of induced pluripotent stem cell-derived fibroblasts (iPSC-FB). iPSC were differentiated into iPSC-FB over 49 days in FGM. On day 49, cells were collected and stained for fibroblast hallmark markers (CD10, CD44, and CD90) and the iPSC characterization marker Tra-1-60. NHDF served as a positive control, iPSC were used as a negative control. Marker expression was analyzed using flow cytometry method. Data is presented as dot plots with color coding distinguishing between the population expressing the marker (orange, in the square) and the population not expressing the marker (blue, outside of the square). *N* = 5.

The iPSC-FB were compared to fibroblasts generated from two different sources, HFDF and NHDF. The differentiation of iPSC into mesodermal cells was performed by adding *L*-ascorbic acid, BMP4, and EGF to the culture environment. After 20 days, cells exhibiting spindle-shaped morphology, characteristic of mesodermal progenitors, emerged from the EB and were successfully subcultured more than ten times. When these differentiated cells were cultured in either FGM or the serum-free CnT-PR-ECM medium, they consistently displayed a fibroblast-like appearance. The study compared the expression of fibroblast-associated surface markers CD10, CD44, and CD90 across iPSC-FB, iPSC, and NHDF. Results showed that NHDF uniformly expressed CD10, CD90, and CD44, without the presence of the stem cell marker Tra-1-60. In contrast, iPSC predominantly expressed Tra-1-60, indicating their stem cell identity. iPSC-FB demonstrated high expression levels of CD10, CD90, and CD44, akin to NHDF, but with slightly elevated levels of Tra-1-60, suggesting a success of the differentiation from iPSC into iPSC-FB (Figure 4).

**Figure 5.**
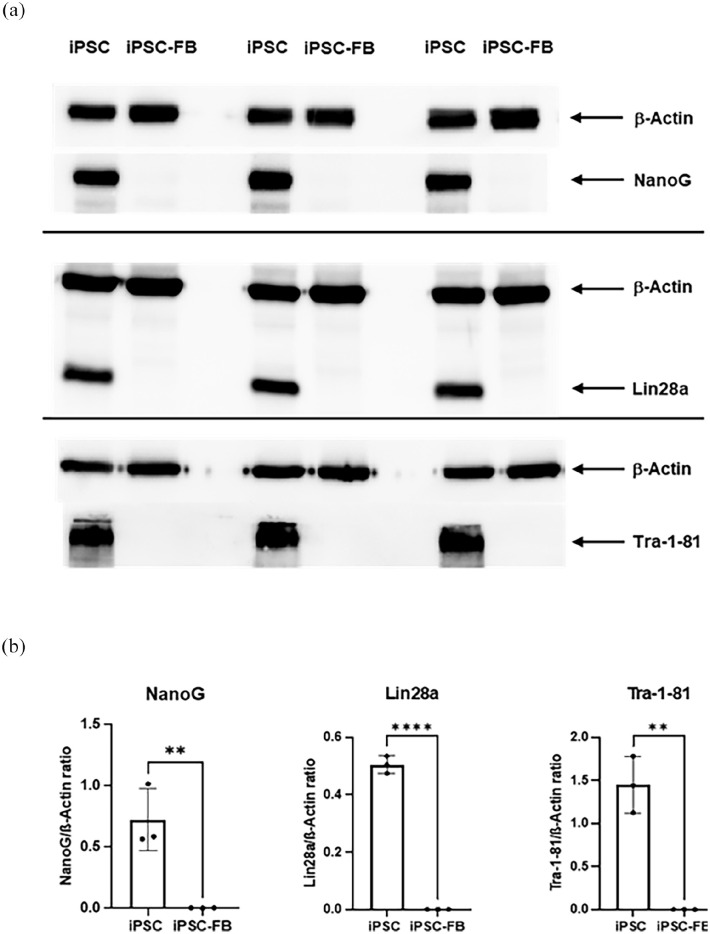
Expression of pluripotency markers in iPSC and iPSC-derived fibroblasts (iPSC-FB). Comparison of pluripotency marker expression between iPSC and iPSC-FB, analyzed via Western blot. Protein expression of Nanog, Lin28a, and Tra-1-81 was evaluated, with β-actin serving as a loading control. (a) Representative Western blot images displaying Nanog, Lin28a, Tra-1-81, and β-actin expression in iPSC and iPSC-FB. (b) Quantitative analysis of Western blot results showing the Nanog/β-actin, Lin28a/β-actin, and Tra-1-81/β-actin ratios. Statistical significance was determined using unpaired t-tests (*p ⩽ 0.05. **p ⩽ 0.01. ***p ⩽ 0.001). These data indicate that iPSC-FB lose expression of key pluripotency markers, confirming their differentiation into fibroblast-like cells. The bar graphs represent mean± *SD, N* = 3.

To further confirm the character of the reprogrammed iPSC and iPSC-FB, western blot analysis was performed to compare the expression of pluripotency markers between iPSC and iPSC-FB. The following well-established pluripotency markers including Tra-1-81, NANOG, and Lin28a were analyzed. The western blot results showed strong signals for these markers in iPSC but not in iPSC-FB, consistent with the loss of pluripotency in differentiated fibroblast cells. This further supports the successful differentiation of the iPSC into iPSC-FB (Figure 5).

**Figure 6.**
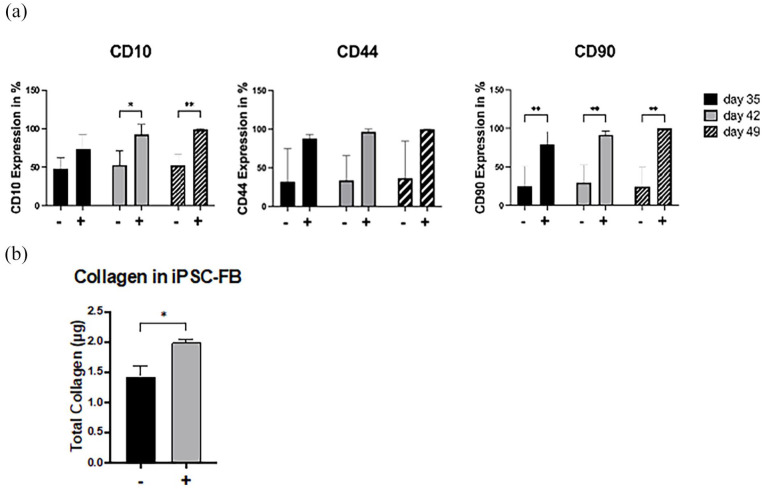
Impact of L-ascorbic acid on iPSC-derived fibroblast characteristics. Influence of L-ascorbic acid on the phenotypic and functional properties of fibroblasts derived from iPSC over a 49-day differentiation period, using fibroblasts differentiation mixtures supplemented with BMP4 and with or without L-ascorbic acid. Marker expression (CD10, CD44, and CD90) and total collagen production were analyzed. (a) The time-course analysis of CD10, CD44, and CD90 expressions between iPSC-FB cultured with (+) and without (−) 0.3 mM L-ascorbic acid at days 35, 42, and 49, determined via flow cytometry. (b) Total collagen production by iPSC-FB on day 49, comparing the presence to the absence of L-ascorbic acid. Statistical significance was assessed using 2-way ANOVA with Sidak’s correction for marker expression and paired t-tests for collagen quantification, with *p-value ⩽0.05 indicating statistical relevance. *N* = 3.

The study also explored how *L*-ascorbic acid influences the differentiation of iPSC into the mesodermal lineage, specifically comparing iPSC-FB differentiated with *L*-ascorbic acid to those differentiated without it. The experimental protocol was identical for both groups, except for the exclusion of *L*-ascorbic acid from one of the differentiation media. Flow cytometry analysis for CD10, CD44, and CD90 was conducted on days 35, 42, and 49 of differentiation. Results indicated a significant enhancement in the expression of fibroblast-associated surface markers CD10, CD44, and CD90 in the presence of *L*-ascorbic acid. By day 49, cells that underwent differentiation with *L*-ascorbic acid showed markedly increased levels of these markers compared to cells differentiated without it (Figure 6(a)). Furthermore, the study assessed differences in collagen secretion between iPSC-FB differentiated under both conditions (Figure 6(b)), providing insights into the biochemical impact of *L*-ascorbic acid on fibroblast differentiation from iPSC. Our findings suggest that *L*-ascorbic acid enhances the stability of the mesodermal lineage and significantly increases collagen secretion in iPSC-FB cells treated with *L*-ascorbic acid compared to those not exposed to it, which is consistent with existing literature.

### Differences in collagen secretion in fibroblasts from different sources and influence of serum in the medium

**Figure 7.**
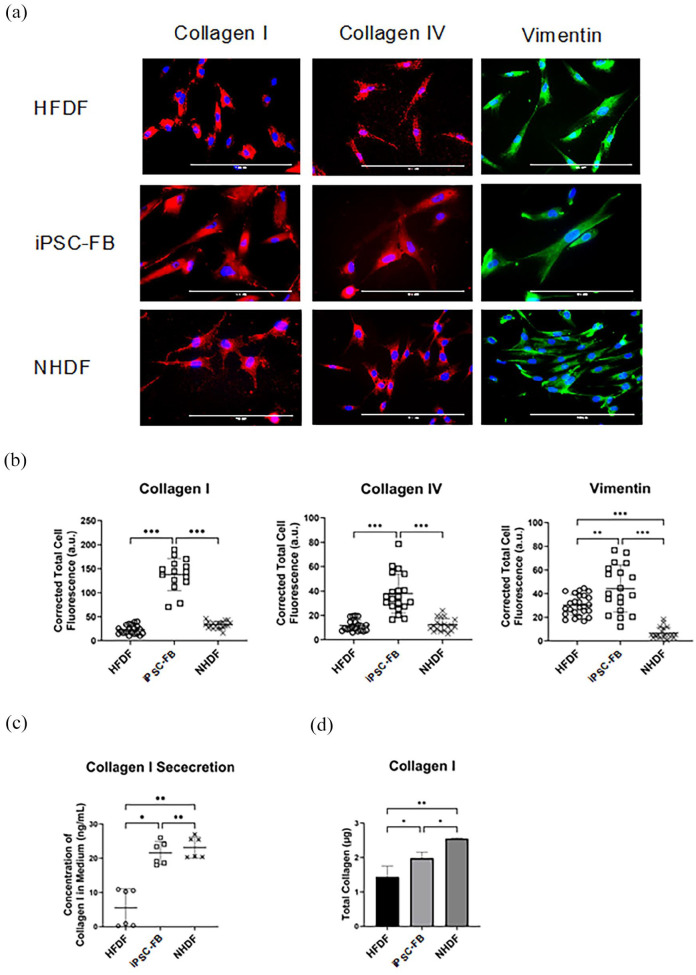
Comparative analysis of collagen synthesis in fibroblasts. Side-by-side comparison of collagen production in iPSC-FB, NHDF, and HFDF. Following iPSC differentiation with L-ascorbic acid for 49 days and subsequent cultivation, samples were analyzed. (a) Microscopic images illustrate the fibroblasts stained for collagen I and IV (red), and vimentin (green), with nuclei counterstained using DAPI (scale bar: 100 µm). Quantitative assessments include (b) corrected total cell fluorescence, (c) secreted collagen I concentration, and (d) total synthesized collagen I, comparing iPSC-FB with NHDF and HFDF. Statistical analysis used one-way ANOVA with Tukey’s correction to identify significant differences (*p ⩽ 0.05. **p ⩽ 0.01. ***p ⩽ 0.001). *N* = 3–6.

A pivotal attribute of fibroblasts is their ability to secrete collagen. This study conducted a comparative analysis to evaluate collagen secretion among iPSC-FB, NHDF, and HFDF. Cells were cultured in FGM for 48 h, followed by IF staining for collagen types I and IV and vimentin. IF staining confirmed the presence of vimentin, a critical protein for cytoskeletal organization, in all fibroblast groups, alongside collagen types I and IV. The intensity of these markers was quantified using the CTCF method, indicating the expression levels of these proteins. Results revealed that all fibroblast variants expressed collagen types I and IV, and vimentin (Figure 7(a)). Quantitative analysis of immunofluorescence staining demonstrated that iPSC-FB had significantly higher expression levels of collagen types I and IV, and vimentin, compared to NHDF and HFDF. These collagens are predominant in the dermis, highlighting the enhanced collagen-producing capacity of iPSC-FB. While NHDF and HFDF showed similar levels of collagen types I and IV expression, HFDF exhibited a significantly higher vimentin expression than NHDF (Figure 7(b)). Furthermore, the collagen I secretion and total collagen I were also investigated between the three cell types; the result showed that iPSC-FB secreted a comparable level of collagen I as the NHDF, confirming the similarity of iPSC-FB and NHDF (Figure 7(c and d)).

**Figure 8.**
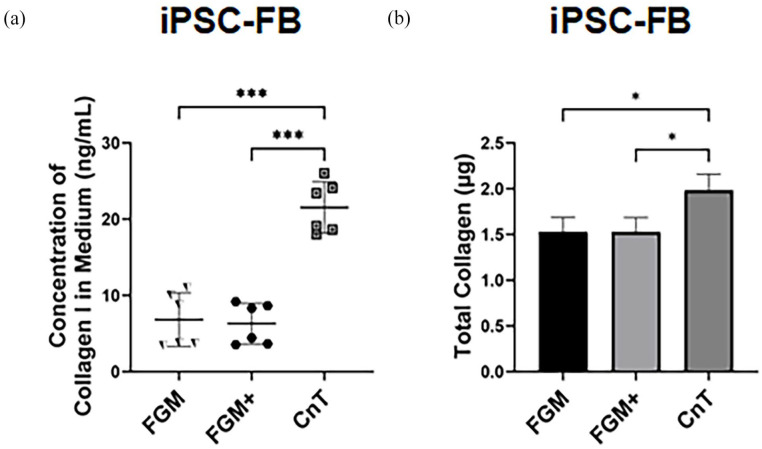
Effect of media on collagen I synthesis in iPSC-derived fibroblasts (iPSC-FB). iPSC-FB were grown in three types of media: FGM, FGM with 0.3 mM L-ascorbic acid (FGM+), and CnT-PR-ECM for 7 days. Following cultivation, both cells and media were analyzed for collagen I production. (a) Collagen I secretion levels in different media. (b) Quantification of total collagen I produced by iPSC-FB across media types. Data were analyzed using one-way ANOVA and Tukey’s post-hoc test (*p ⩽ 0.05. **p ⩽ 0.01. ***p ⩽ 0.001). *N* = 3–6.

Furthermore, the effect of serum in cell culture media on iPSC-FB was investigated. The results of the enzyme-linked immunosorbent assay performed on iPSC-FB showed that the concentration of type I collagen secreted by fibroblasts into the culture medium was more than twice as high in CnT-PR-ECM, a serum-free medium, compared to FGM and FGM supplemented with *L*-ascorbic acid (FGM+). Additionally, the total amount of all soluble collagens, both intracellular and secreted, was quantified using a quantitative collagen assay. The assay confirmed that fibroblasts cultured in CnT-PR-ECM had the highest collagen levels (Figure 8(a and b)). The results suggest that iPSC- FB are a promising source for constructing self-assembled skin models due to their superior collagen secretion and extra-cellular matrix-forming capabilities compared to HFDF. Together with the defined medium, it is possible to exclude the animal component while increase higher collagen synthesis rate of the cells.

### Characterization of iPSC derived keratinocytes in comparison to hair-follicle and human foreskin derived keratinocytes

**Figure 9.**
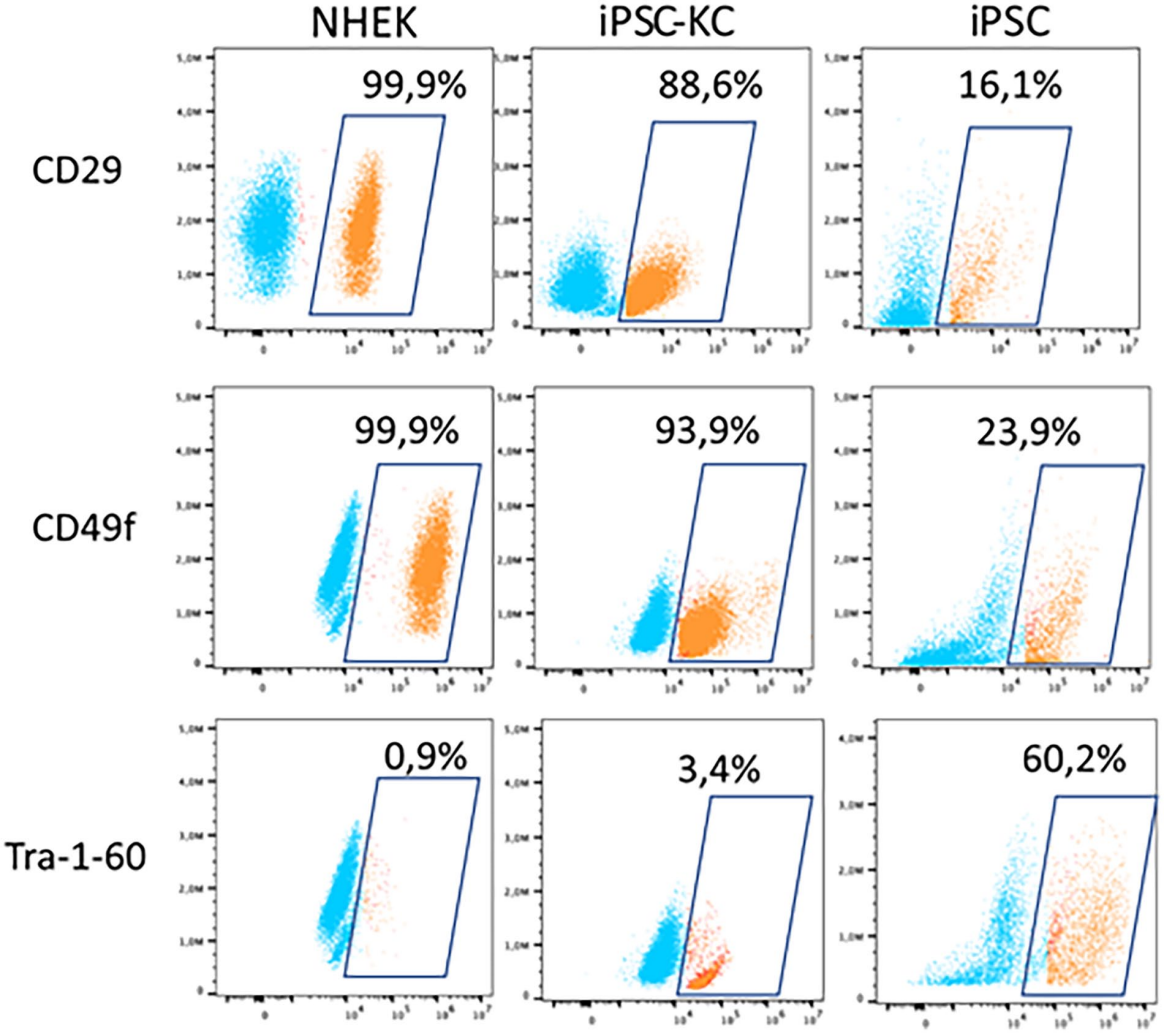
Flow cytometry analysis of iPSC-derived keratinocytes (iPSC-KC). iPSC-KC were differentiated using retinoic acid and BMP4. On day 35, cells were stained for keratinocyte markers (CD29 and CD49f) and iPSC marker Tra-1-60 and analyzed by flow cytometry. NHEK and undifferentiated iPSC served as positive and negative controls, respectively. Dot plots display populations expressing markers (orange, within square) versus non-expressing populations (blue, outside square). *N* = 5.

To generate keratinocytes, we adjusted the differentiation protocol to promote differentiation toward the ectodermal lineage. This modification included the addition of retinoic acid and BMP4 to the culture medium. iPSC-KC were differentiated from iPSC. They were then characterized and compared to their counterparts isolated from human foreskin, the NHEK. A flow cytometry analysis was conducted to examine the cell surface markers CD29 and CD49f, which are part of the integrin family and associated with keratinocytes. The results revealed a high expression of both markers in iPSC-KC and NHEK compared to iPSC. Both types of keratinocytes expressed less than 5% of Tra-1-60, a pluripotency marker of stem cells, indicating the successful differentiation of iPSC into keratinocytes (Figure 9).

**Figure 10.**
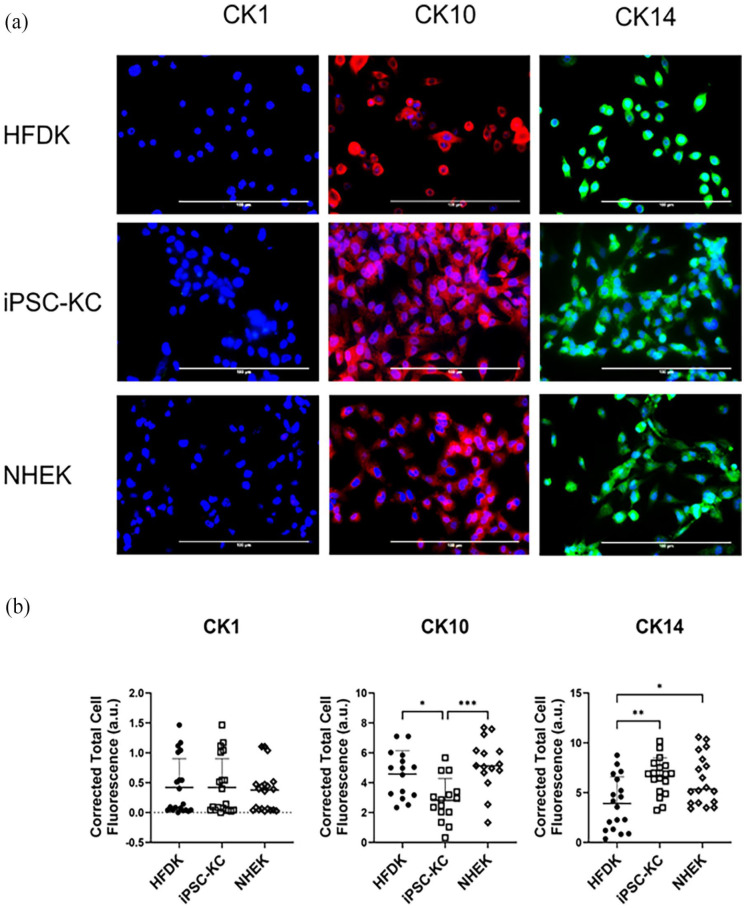
Comparative analysis of keratinocyte phenotypes derived from iPSC, normal human epidermis, and hair follicles. Characterization of iPSC-KC relative to NHEK and HFDK. Differentiation of iPSC into iPSC-KC was achieved using medium supplemented with retinoic acid and BMP4. (a) Immunofluorescence microscopy showcases the expression of keratinocyte markers: cytokeratin 1 (CK1-red), cytokeratin 10 (CK10-red), and cytokeratin 14 (CK14-green) across iPSC-KC and NHEK samples. Nuclei staining was performed with DAPI to facilitate cell count and structure observation. Scale bar of 100 µm for size comparison is indicated. (b) Quantitative analysis of keratinocyte marker expression via CTCF among iPSC-KC, NHEK, and HFDK. Statistical significance was determined using one-way ANOVA complemented by Tukey’s post-hoc test for multiple comparisons, highlighting differences in marker expression (*p ⩽ 0.05. **p ⩽ 0.01. ***p ⩽ 0.001).

The study employed immunostaining techniques with various cytoskeletal markers to compare the expression levels of these markers among keratinocytes derived from different sources. To quantify the expression of these markers, the CTCF method was utilized. The findings revealed significant differences in the expression levels of CK10, a marker associated with keratinocyte differentiation. Specifically, iPSC-KC exhibited the lowest level of CK10, suggesting a less differentiated state than other sources. However, the level of CK14 in iPSC-KC was found to be comparable to that in NHEK, indicating a closer resemblance between iPSC-KC and NHEK in terms of their cytoskeletal marker profile. This suggests that while iPSC-KC may exhibit a lower differentiation state as indicated by CK10 levels, their CK14 expression levels suggest a fundamental similarity to normal keratinocytes regarding keratinocyte identity and basic epithelial characteristics (Figure 10).

### Development and characterization of a xeno-free self-assembled skin model from iPSC-derived cells

**Figure 11.**
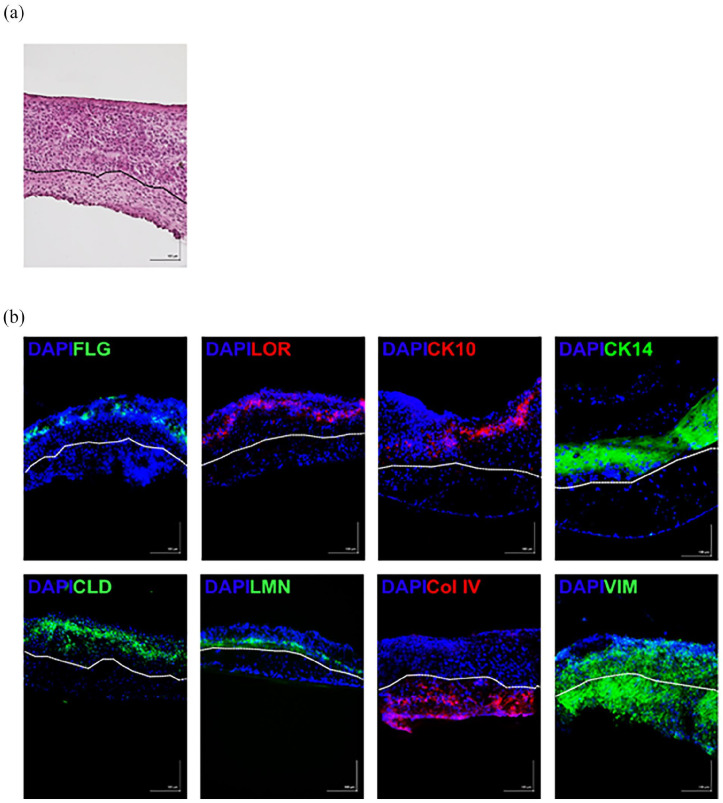
Characterization of self-assembled skin models (SASM). Cryosectioned SASM were mounted on microscope slides and stained with either H&E for general morphology (a) or immunofluorescence for specific markers (b). Immunofluorescence staining targeted epidermal markers including filaggrin (FLG-green), loricrin (LOR-red), cytokeratin 10 (CK10-red), and cytokeratin 14 (CK14-green). Tight junctions between cells were visualized using claudin (CLD-green) staining, and the dermal-epidermal junction was demarcated by laminin (LMN-green). The dermis was marked by collagen type IV secreted by the iPSC-FB. Cytoskeleton is indicated by vimentin (VIM-green). Nuclei were counterstained with DAPI. Scale bar = 100 µm. *N* = 3.

As the final stage of the experiment, full-thickness skin equivalents were generated using iPSC-FB and iPSC-KC. Initially, iPSC-FB were seeded onto a porous cell membrane and cultured in CnT-PR-ECM for 14 days. During this period, the fibroblasts secreted extracellular matrix components. On the 15th day, iPSC-KC were seeded onto the fibroblast layer. After 24 h, the constructs were transitioned to the air-liquid interface. The skin models were cultured for an additional 10 days. Examination of hematoxylin and eosin stained cryosections of the skin equivalents via light microscopy showed the formation of separate epidermal and dermal layers. iPSC-KC formed a stratified epidermis with a visible stratum corneum, while iPSC-FB secreted collagen effectively, creating a dermal layer without additional collagen supplementation. The entire culturing process was carried out using a serum-free medium (Figure 11(a)).

The development of the first self-assembled skin model from iPSC-derived cells marks a significant advancement. Immunofluorescence staining has verified the presence of tight junction proteins, such as claudin, across dermal and epidermal layers, highlighting its critical role in regulating paracellular permeability. Notably, the expression of claudin was especially pronounced within the epidermal layer of the SASM. Additionally, the SASM showcased the presence of filaggrin and loricrin, key proteins in filament aggregation, predominantly expressed in the stratum corneum and located at the outermost layer of the epidermis.

The model also demonstrated positive staining for CK10, indicating the onset of keratinocyte differentiation within the suprabasal layers of the epidermis. Positive staining for CK14, indicative of the basal layer keratinocytes, further underscores the cellular complexity of the skin model. Laminin, which is known to be the dermal epidermal junction is also positive in the SASM. Moreover, the distinct presence of collagen IV within the dermis layer confirms collagen deposition by iPSC-FB, while the presence of vimentin in both epidermal and dermal layers aligns with expectations, reflecting the robust structural integrity of the SASM (Figure 11(b)). Furthermore, the endeavor to construct SASM using cells derived from hair follicles has proven unsuccessful. The HFDF were incapable of producing a sufficient quantity of collagen to give rise to the dermis layer, and only the epidermis was visible (data not shown). This observation corroborates the finding that HFDF secrete significantly less collagen compared to induced iPSC-FB.

### Functionality of the self-assembled skin model

The self-assembled skin model, free from xenobiotic compounds, was cultured for 24 days to secrete an extracellular matrix. In comparison to conventional skin models that are cultivated with animal-derived compounds and an artificial extracellular matrix made of collagen or a similar scaffold, the SASM requires a longer cultivation period. Therefore, the aim is to confirm the functionality and viability of the cells in the epidermal and dermal layers.

**Figure 12.**
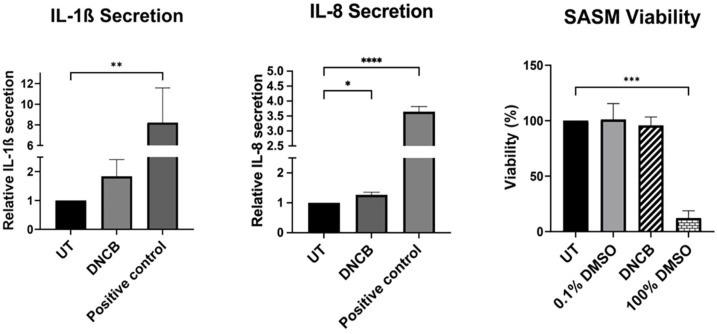
Effects of DNCB on SASM cell viability and cytokine secretion. SASM cells were incubated with DNCB for 24 h to assess cellular responses. Cell viability was determined via the MTT assay, comparing untreated cells (UT), a vehicle control (0.1% DMSO), and a positive control (100% DMSO). Additionally, cytokine analysis involved measuring IL-8 and IL-1β levels in the culture medium using ELISA, with comparisons made to untreated and positive control samples (50 ng/mL TNF-α and 50 ng/mL IL-1β). This figure illustrates DNCB’s impact on SASM cells, highlighting both metabolic activity and inflammatory response. *N* = 3.

The secretion levels of IL-1β and IL-8 cytokines were quantitatively measured using an ELISA assay, while cell viability was assessed through the MTT assay. The skin models underwent treatment with the potent skin sensitizer DNCB, which was solubilized in DMSO. For comparative analysis, an untreated control and a positive control treated with an inflammatory cytokine cocktail were included. In the MTT assay, to establish benchmarks for cell viability, 100% DMSO served as a positive control, whereas 0.1% DMSO was utilized as a vehicle control (Figure 12).

Following DNCB application, there was a noticeable trend toward elevated IL-1β secretion, alongside a significant surge in IL-8 secretion. This pattern underscores the sensitivity of the self-assembled skin models to the skin sensitizer DNCB. It should be noted that undifferentiated iPSC, iPSC-FB, and iPSC-KC did not exhibit an IL-1β response when exposed to DNCB (data not shown). Moreover, MTT assay results indicated robust metabolic activity across the untreated control, vehicle control, and DNCB-treated samples. This demonstrates that the SASM maintains high viability over extended culture periods and possesses the capability to actively respond to sensitizing agents. Thus, these findings affirm the functional integrity and ability of the SASM to recognize sensitizer.

In conclusion we suggest a xenogeneic-free culture method for self-assembled skin models.

## Discussion

The European movement advocating reduced animal testing underscores the ethical imperative in research. Human reconstructed skin models (RHS) are gaining prominence as a superior alternative, addressing ethical concerns and providing more relevant and reliable data than traditional animal models. RHS has a customizable nature that allows for tailored characteristics, and their accessibility and scalability offer practical advantages, emphasizing their pivotal role in advancing research while minimizing reliance on animal testing.

The current RHS relies on animal-derived components such as FBS or bovine collagen type I, which are esteemed for their reliability and well-understood characteristics. However, concerns have emerged regarding the variability in animal sourcing, ethical considerations surrounding animal welfare in material procurement for research purposes, and the accuracy of these materials in representing human biology faithfully. Additionally, extensive discussions have underscored the presence of contaminants in FBS, such as non-human *N*-glycolylneuraminic acid, possibly impeding the translation of pre-clinical findings into clinical applications.^16–18^ Furthermore, studies have reported that bovine collagen has the potential to elicit an immune response in humans.^19,20^ Despite less scientific scrutiny in comparison to FBS concerning the use of bovine collagen in research, the process of obtaining collagen involves animal pain and suffering.

Given this evidence, it is imperative to seek an alternative for dermatological research that is both ethical and sustainable. Therefore, the aim was to develop a xeno-free alternative known as SASM, wherein fibroblasts produce the dermis through collagen secretion. To achieve a sustainable and ethically sound cell source, HFDK were obtained from donors and reprogrammed into iPSC. Subsequently, these iPSC were differentiated into fibroblasts (iPSC-FB) and keratinocytes (iPSC-KC).

The selection of HFDK for reprogramming into iPSC was motivated by their accessibility, ease of cultivation, and previously documented success in reprogramming HFDK or NHEK into iPSC, as indicated in the relevant literature demonstrated.^21–23^ While the Sendai virus was chosen for its high efficiency and non-integrative nature, alternative vectors with an improved safety profile should be considered.

Upon reprogramming process, a noteworthy morphological transformation of HFDK into colony-like structures was observed after a certain duration, aligning with findings suggesting that such morphological changes signify the gradual transition of cells into a stem cell state.^24,25^ In addition to the morphological alterations, the iPSC colonies derived from HFDK exhibit the expression of key stem cell markers, including NANOG, SOX2, SSEA4, Tra1-60, and Oct3/4. This pattern of marker expression is consistent with established characteristics of pluripotent stem cells, as confirmed by studies utilizing these specific markers for the identification of stem cell colonies.^26–28^ Moreover, these colonies also do not express CK1, CK10, and CK14, well known keratinocyte characterization markers which also align with other studies where the iPSC were reprogrammed from HFDK or NHEK.^29,30^ Furthermore, expression of pluripotency markers by the iPSC was further confirmed using qPCR analyzing SOX2, NANOG, POU5F1, DNMT3B, TERT, UTF1, DPPA5, IDO1, KLF4, and KLF17 gene expression and the western blot analyzing NANOG, Lin28a, and Tra-1-81 protein expression. NANOG and POU5F1 (OCT4) are critical transcription factors that, along with SOX2, form a core network preserving the undifferentiated state of stem cells. Lin28a plays a significant role in reprogramming and regulating stem cell fate. Surface marker Tra-1-81 is commonly used to identify undifferentiated iPSC. Additionally, KLF4 and KLF17 contribute to reprogramming and regulating pluripotency and cell proliferation. DNMT3B, a DNA methyltransferase, establishes epigenetic marks necessary for gene regulation, while TERT maintains telomere length for long-term self-renewal. UTF1 and DPPA5 help regulate gene expression and cell survival, supporting the pluripotent state. IDO1 aids in immune tolerance, preventing rejection in therapeutic applications. Together, these genes and markers are central to maintaining the essential properties of iPSC. The robust expression of iPSC characterization markers and the absence of keratinocyte characterization markers serve as a crucial indicator of the successful reprogramming of HFDK into iPSC.

Following iPSC differentiation, iPSC-FB were generated using protocols adapted from other studies,^12,31^ which are foundational in understanding the differentiation process. Culturing iPSC as EB is a widely used method to promote spontaneous differentiation. EBs are three-dimensional aggregates of iPSC that can differentiate into cells of all three germ layers, which are ectoderm, mesoderm, and endoderm. They represent a simplified model of early embryonic development. Within the EB microenvironment, iPSC are driven toward spontaneous differentiation, influenced by their localization within the EB. Furthermore, differentiation toward specific lineages can be achieved by incorporating differentiation factors.

The differentiation of iPSC toward the mesodermal lineage was achieved by supplementing the culture with *L*-ascorbic acid, BMP4, and EGF. *L*-ascorbic acid supplementation, explored for its documented ability to enhance fibroblast maturation, draws upon foundational work by other studies,^32,33^ highlighting its role in cellular maturation processes. This supplementation resulted in accelerated differentiation and sustained expression of fibroblast markers in iPSC, and higher collagen synthesis than the iPSC-FB differentiated without *L*-ascorbic acid. Numerous studies highlight the efficacy of *L*-ascorbic acid in enhancing mesenchymal cell reprogramming into iPSC by modulating histone demethylases.^34–36^ This underscores its universal applicability across species, while investigations into the role of *L*-ascorbic acid in iPSC differentiation into fibroblasts are limited. *L*-ascorbic acid serves as a cofactor for enzymes crucial in collagen biosynthesis, essential for extracellular matrix integrity, and upregulates TGFβ1-induced genes related to fibroblast differentiation and function.^37,38^ Moreover, *L*-ascorbic acid is a crucial cofactor for prolyl-4-hydroxylase and lysyl hydroxylase, enzymes essential in collagen maturation.^39^ Hence, *L*-ascorbic acid could potentially enhance iPSC differentiation into fibroblasts by promoting fibroblast-specific marker expression and collagen synthesis, facilitating their maturation.

Furthermore, iPSC-FB differentiated with *L*-ascorbic acid exhibited increased collagen secretion compared to cells deprived of *L*-ascorbic acid throughout the differentiation process. This effect likely stems from the capacity of *L*-ascorbic acid to enhance TGFβ1-induced expression of genes related to collagen synthesis and αSMA stress fiber formation, thereby promoting the synthesis and deposition of collagens type I and IV.^40^ The broader literature supports these findings, emphasizing the multifaceted role of *L*-ascorbic acid in the collagen biosynthesis, including its impact on mRNA levels of collagen and related enzymes,^41^ and its antioxidant properties which may further enhance fibroblast function and collagen stability.^42,43^

The collagen synthesizing ability of iPSC-FB was further characterized by comparing them with NHDF obtained from juvenile foreskin and HFDF. The results revealed that iPSC-FB can synthesize collagen type I and IV similarly to NHDF, whereas HFDF exhibited inferior performance in this regard. Moreover, the iPSC-FB comparably express the typical fibroblasts surface markers, CD10, CD44, and CD90 as the NHDF. These findings are consistent with those of previous studies.^31,44^

Additionally, a difference in fibroblast collagen secretion when cultured in the presence or absence of serum in the medium was observed. Fibroblasts are typically cultured in DMEM supplemented with fetal bovine serum, which provides signaling molecules that promote ECM synthesis, including collagen. Furthermore, serum supplementation is expected to enhance fibroblast proliferation, thereby increasing collagen production and secretion.^45,46^ Our findings indicate that iPSC-FB cultured in CnT-PR-ECM, a medium specifically designed to promote extracellular matrix protein secretion and free from animal or human components, exhibit higher collagen I compared to those cultured in FGM, which contains 10% serum.

The differentiation of iPSC-KC was achieved by implementing a protocol adapted from other studies.^12,15^ In the presence of retinoic acid and BMP4, the cells were successfully differentiated. Characterization of cells indicated the expression of keratinocyte-specific markers (CD29, CD49f, as well as different cytokeratins). Remarkably, the results demonstrated that these cells exhibited characteristics comparable to NHEK and HFDK underscoring the success and specificity of the differentiation process. The results correspond to the findings from other studies.^47,48^

The combination of retinoic acid and BMP4 was strategically selected to create a robust and physiologically relevant skin model. The development of SASM involved leveraging the collagen secretion capabilities of iPSC-FB and the strategic seeding of iPSC-KC on top of the self-generated dermal layer. Several studies have successfully employed a similar approach with neonatal fibroblasts or foreskin fibroblasts.^49–52^ Remarkably, the results of the SASM experiment demonstrated the feasibility of iPSC-FB independently secreting collagen and forming a dermal layer with an extracellular matrix, without relying on animal components, further affirming the comparable functionality of iPSC-FB and NHDF. Additionally, iPSC-KC, when seeded onto the dermal layer, underwent successful differentiation into a fully stratified epidermis, paralleling the expectations from the epidermis generated using NHEK as shown in the mentioned studies. The characterization of the SASM revealed an epidermal layer expressing laminin, loricrin, filaggrin, CK10, and CK14, along with tight junction proteins indicated by claudin. This characterization is consistent with the features observed in RHS models derived from NHEK or HFDK.^13,47,53^ Moreover, a crucial functionality assessment was conducted using the MTT assay on SASM, and the results unequivocally demonstrated its functional competence.

Moreover, a crucial functionality assessment was conducted by measuring the IL-8 and IL1b secretion from the SASM after being treated with DNCB. IL-8 and IL-1β are known cytokines that can be used to measure the response of keratinocytes to the skin sensitizers.^54^ The results unequivocally demonstrated its functional competence. Furthermore, despite the long cultivation time of the SASM, the MTT result reveals the unchanged viability even with the treatment showcasing that the SASM is not fragile, and the cells remain metabolically active. This finding further underscores the potential utility of SASM in various applications.

To our knowledge, this is the first self-assembled skin models where iPSC-derived cells were used as a source. The study demonstrates the remarkable progress made in exploiting the potential of fibroblasts for skin tissue engineering. The successful differentiation of iPSC into keratinocytes, resembling NHEK features, and the generation of SASM underscore the collaborative ability of iPSC-FB and iPSC-KC to form functional skin structures. SASM stands out for its distinct ethical advantages, as it does not rely on animal components. Every component and cell within the SASM were generated xeno-free, offering a groundbreaking model that can be derived from a single donor. The flexibility of the SASM model allows for genetic modifications, such as knocking out specific genes in iPSC-FB or iPSC-KC to simulate disease conditions. This opens up possibilities for using the model not only to study the pathophysiology of such conditions but also to screen potential therapeutic agents that could mitigate the symptoms or underlying causes of these disorders.

Our results contribute to the regenerative medicine field, emphasizing the consistency and ethical implications of utilizing iPSC-derived skin models.
